# Real-world study of trastuzumab and pertuzumab combined with chemotherapy in neoadjuvant treatment for patients with HER2-positive breast cancer

**DOI:** 10.1097/MD.0000000000030892

**Published:** 2022-10-07

**Authors:** Xiangmin Ma, Xiangmei Zhang, Xinping Zhou, Xiaofei Ren, Xindi Ma, Weifang Zhang, Ruiling Yang, Tao Song, Yunjiang Liu

**Affiliations:** a Department of Breast Center, Fourth Hospital of Hebei Medical University, Shijiazhuang City, Hebei, China; b Research Center, Fourth Hospital of Hebei Medical University, Shijiazhuang City, Hebei, China; c Hebei Provincial Key Laboratory of Tumor Microenvironment and Drug Resistance, Hebei Medical University, Shijiazhuang City, Hebei, China; d Department of Breast Surgery, Handan Central Hospital, Handan City, Hebei, China.

**Keywords:** breast cancer, human epidermal growth factor receptor 2 (HER2), neoadjuvant treatment, pathological complete response (pCR), pertuzumab

## Abstract

Clinical trials have shown that trastuzumab (H) and pertuzumab (P) combined with chemotherapy as neoadjuvant therapy increased pathological complete response (pCR) rate of patients with human epidermal growth factor receptor 2 (HER2)-positive breast cancer. However, date in China in the real world are currently limited.

Clinical data from patients with HER2-positive breast cancer who received HP combined with chemotherapy as neoadjuvant therapy at 2 institutions from March 2019 to February 2022 were retrospectively analyzed. Adverse reactions were evaluated using CTCAE version 5.0. The primary endpoint was total pathologic complete response (tpCR; ypT0/isypN0), and the secondary endpoints were breast pathologic complete response (bpCR; ypT0/is) and axillary pathologic complete response (apCR; ypN0). Factors influencing tpCR were also analyzed.

A total of 302 patients were included in the analysis, of which 145 were treated with H + P + taxane + carboplatin (TcbHP), 94 with H + P + taxane (THP) and 63 with sequential anthracycline and cyclophosphamide, followed by H + P + taxane (AC-THP). The overall tpCR rate was 64.9%, and those of TcbHP, THP, and AC-THP were 73.1%, 52.1%, and 65.1%, respectively. The tpCR rate of the hormone receptor (HR) negative group (80.3%) was higher than that of the HR positive group (52.1%). The overall bpCR rate was 73.5% and the apCR rate was 75.8%. In the univariate analysis, HR, HER2 status and treatment regimen were related factors that affected tpCR. In the multivariate analysis, HR, HER2 status and treatment regimen were independent predictors of tpCR (*P* < .001, *P* < .001 and *P* = .009). The levels 3 and 4 toxicities rates of TcbHP were slightly higher than those of THP and AC-THP.

HP combined with chemotherapy has achieved a high pCR rate. The TcbHP regimen had the highest pCR. HR-negative tumors demonstrated a higher pCR. HR, HER2 status and treatment regimen were independent predictors of tpCR. The adverse reactions are controllable.

## 1. Introduction

According to the latest data on the 2020 global burden of cancer, the number of new breast cancer cases rapidly reached 2.26 million, and breast cancer becomes the world’s largest cancer.^[1]^ The overexpression of human epidermal growth factor receptor 2 (HER2) (HER2-positive) accounts for 15% to 25% of patients with breast cancer.^[2,3]^ This type is associated with aggressive clinical phenotypes and poor outcomes.^[4]^ The application of targeted drugs significantly improved the survival rate of HER2-positive breast cancer. Neoadjuvant therapy greatly improves tumor resectability and breast conservation rate. The pathologic complete response (pCR) can be used as a reliable prognostic indicator; achieving a pCR will lead to a longer survival.^[5]^ The NeoSphere study^[6]^ confirmed that in neoadjuvant therapy, compared to chemotherapy plus trastuzumab (H), the dual anti-HER2 blockade strategy with pertuzumab (P) and trastuzumab combined with chemotherapy increased pCR rates. The subsequent KRISTINE^[7]^ and TRAIN-2^[8]^ studies further confirmed the efficacy and safety of HP combined with different chemotherapy drugs.

Pertuzumab was approved in December 2018 in China, and in March 2019, its clinical application was officially started. A small number of patients used pertuzumab due to the high cost. Therefore, prices were reduced in January 2020, and were officially included in the country’s health care, driving the Chinese standardization process for the treatment of HER2-positive breast cancer. Currently, few data have been reported in China on HP combined with different chemotherapy drugs as neoadjuvant therapy for HER2-positive breast cancer in the real world.

With the above background, this study reported the real-world clinical efficacy and adverse reactions of HP combined with chemotherapy as neoadjuvant therapy in patients with HER2-positive breast cancer treated in two institutions from March 2019 to February 2022 in Hebei Province of North China. This study aimed to provide real-world clinical data on the application of pertuzumab and trastuzumab in Chinese patients.

## 2. Patients and Methods

### 2.1. Study design and patients

We retrospectively analyzed data of patients with HER2-positive breast cancer who received HP combined with chemotherapy as neoadjuvant therapy and underwent surgery at the Breast Cancer Center of the Fourth Hospital of Hebei Medical University and Handan Central Hospital from March 2019 to February 2022. The inclusion criteria were as follows: core-needle biopsy-confirmed diagnosis of HER2-positive breast cancer, complete neoadjuvant treatment, ≥4 cycles of dual-target combination therapy, with surgery, and complete pathological data. The exclusion criteria were as follows: metastatic or recurrent breast cancer as the first diagnosis, bilateral breast cancer, occult breast cancer, incomplete clinicopathological information, and without surgery.

These studies were approved by the Ethics Committee of the Fourth Hospital of Hebei Medical University and the Ethics Committee of the Handan Central Hospital (Ethics Number:2021KY262). Informed consent to data collection was obtained from all the participants involved.

### 2.2. Biomarker detection

The expression of estrogen receptor (ER) and progesterone receptor (PR) was immunohistochemically analyzed according to the 2010 American Society of Clinical Oncology/College of American Pathologists guidelines.^[9]^ The status of HER2 was determined by the 2018 HER2 guidelines.^[10]^ The HER2 status was first determined by immunohistochemistry (IHC), and patients with IHC 3 + tumors were diagnosed as HER2-positive; for patients with HER2 IHC 2 + tumors, HER2 was identified positive by fluorescence in situ hybridization (FISH). Tumors were classified as hormone receptor (HR) positive if ≥1% of tumor cells stained for ER and/or PR. Ki-67 levels used the 14% cutoff (>14% vs ≤14%).

### 2.3. Scheme of treatment

All patients received a standard preoperative therapy regimen according to the National Comprehensive Cancer Network guidelines for invasive breast cancer and classic clinical trials.^[6,11]^ The treatment regimens were as follows: paclitaxel or docetaxel + carboplatin + trastuzumab + pertuzumab (TcbHP); paclitaxel or docetaxel + trastuzumab + pertuzumab (THP); anthracycline + cyclophosphamide (AC) followed by paclitaxel, or docetaxel + trastuzumab + pertuzumab (THP) (AC-THP). The doses of treatment drugs were based on the NCCN guidelines.

Surgery was performed 14 to 21 days after preoperative therapy. The surgery types included total mastectomy, breast-conserving surgery, and breast reconstructions depending on the patient’s condition and intention. Sentinel lymph node biopsy was performed in selected patients.

### 2.4. Efficacy evaluation

The treatment efficacy was evaluated by magnetic resonance imaging (MRI) of the breast according to RECIST1.1.^[12]^ Clinical efficacy evaluations were classified as complete response (CR), partial response (PR), stable disease (SD), and progressive disease (PD).

The primary endpoint was the rate of total pathologic complete response (tpCR) in the breast and lymph nodes (ypT0/isypN0, the absence of invasive cancer in the breast and axillary lymph nodes, regardless of the remaining ductal carcinoma in situ in the primary tumor). The secondary endpoints were the rate of pCR in the breast (breast pathological complete response [bpCR]) (ypT0/is) and axillary pathologic complete response (apCR) (ypN0). The Miller-Payne (MP) classification system or the residual breast cancer burden (RCB) have been used to assess pCR.^[13,14]^

Adverse events (AEs) were categorized according to National Cancer Institute Common Terminology Criteria for Adverse Events (NCI-CTCAE) version 5.0. They were divided into grades 1, 2, 3, and 4.

### 2.5. Statistical analysis

All statistical analyses were conducted using the Statistical Package for the Social Sciences (SPSS) version 21.0 (SPSS Inc, Chicago, IL). Chi-squared was used for the comparison of categorical variables. Univariate and multivariate analysis was performed using logistic regression for clinicopathological parameters associated with tpCR. All analyzes used two-tailed p-values and a *P* value <.05 was considered statistically significant. 95% confidence interval (CI) was calculated to evaluate the additive interaction.

## 3. Results

### 3.1. Patient characteristics

**Figure 1.**
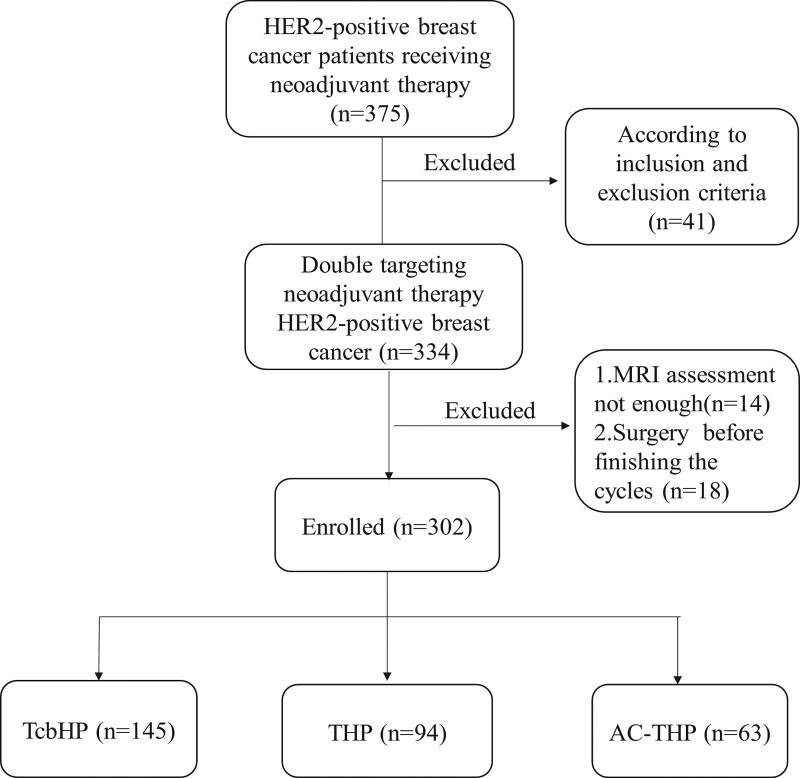
Flow diagram of patients.

From March 2019 to February 2022, a total of 375 patients were enrolled in the study, and 73 patients were excluded because they did not meet the selection criteria. Therefore, a total of 302 patients were evaluable for analysis. The flow diagram of the patients is shown in Figure 1. The median age was 52 (range, 22–74) years. Infiltrating ductal carcinoma was present in 281 (93%) patients. Of the 302 patients, 145 were treated with the TcbHP regimen, 94 with THP, and 63 with AC-THP. Patients with clinical stage III status accounted for 58.3% (176/302). Breast-conserving surgery was successfully performed in 49 (16.2%) patients. The clinical and pathological characteristics of the evaluable patients are outlined according to treatment regimens (Table 1). No differences were found among the treatment regimens (*P* > .05).

**Table 1:** Clinical and pathological characteristics of patients with HER2-positive breast cancer treated with different neoadjuvant therapies (N [%]).

Characteristics	Total (%)	TcbHP (%)	THP (%)	AC-THP (%)	*P*
	N = 302	N = 145	N = 94	N = 63	
**Age (yrs**)					.265
** Median age (range**)	52 (22–74)	52 (27–73)	54 (26–74)	49 (22–70)	
** ≤50**	137 (45.4)	67 (46.2)	37 (39.4)	33 (52.4)	
** >50**	165 (54.6)	78 (53.8)	57 (60.6)	30 (47.6)	
**Menopause**					.100
** No**	139 (46.0)	68 (46.9)	36 (38.3)	35 (55.6)	
** Yes**	163 (54.0)	77 (53.1)	58 (61.7)	28 (44.4)	
**Clinical T stage**					.907
** cT1**	24 (7.9)	9 (6.2)	10 (10.6)	5 (7.9)	
** cT2**	187 (61.9)	89 (61.4)	59 (62.8)	39 (61.9)	
** cT3**	42 (13.9)	22 (15.2)	11 (11.7)	9 (14.3)	
** cT4**	49 (16.2)	25 (17.2)	14 (14.9)	10 (15.9)	
**Clinical N stage**					.331
** cN0**	21 (7.0)	11 (7.6)	4 (4.3)	6 (9.5)	
** cN1**	145 (48.0)	65 (44.8)	46 (48.9)	34 (54.0)	
** cN2**	57 (18.9)	29 (20.0)	22 (23.4)	6 (9.5)	
** cN3**	79 (26.2)	40 (27.6)	22 (23.4)	17 (27.0)	
**Clinical stage**					.554
** II**	126 (41.7)	59 (40.7)	37 (39.4)	30 (47.6)	
** III**	176 (58.3)	86 (59.3)	57 (60.6)	33 (52.4)	
**Histological classification**					.314
** G2**	267 (88.4)	124 (85.5)	86 (91.5)	57 (90.5)	
** G3**	35 (11.6)	21 (14.5)	8 (8.5)	6 (9.5)	
**ER**					.932
+	149 (49.3)	73 (50.3)	45 (47.9)	31 (49.2)	
–	153 (50.7)	72 (49.7)	49 (52.1)	32 (50.8)	
**PR**					.466
+	129 (42.7)	67 (46.2)	36 (38.3)	26 (41.3)	
–	173 (57.3)	78 (53.8)	58 (61.7)	37 (58.7)	
**HR**					.694
+	165 (54.6)	81 (55.9)	48 (51.1)	36 (57.1)	
–	137 (45.4)	64 (44.1)	46 (48.9)	27 (42.9)	
**HER2**					.072
** IHC 2+/FISH +**	36 (11.9)	11 (7.6)	16 (17.0)	9 (14.3)	
** IHC 3+**	266 (88.1)	134 (92.4)	78 (83.0)	54 (85.7)	
**Ki-67**					.573
** ≤14%**	11 (3.6)	4 (2.8)	5 (5.3)	2 (3.2)	
** >14%**	291 (96.4)	141 (97.2)	89 (94.7)	61 (96.8)	

AC-THP = anthracycline + cyclophosphamide sequentially taxane, or docetaxel + H + P, ER = estrogen receptor, FISH = fluorescence in situ hybridization, HR = hormone receptor, HER2 = human epidermal growth factor receptor 2, IHC = immunohistochemistry, PR = progesterone receptor, TcbHP = taxane or docetaxel + carboplatin + trastuzumab(H) + pertuzumab (P), THP = taxane or docetaxel + H + P.

### 3.2. Evaluation of efficacy of neoadjuvant therapy

Magnetic resonance imaging was used to assess clinical efficacy according to RECIST 1.1. Of the 302 patients, 59.3%, 32.5%, and 8.3% achieved CR, PR, and SD, respectively. No patient had PD. Among the patients, 196 (64.9%) achieved total pCR; 222 (73.5%) achieved bpCR, and 229 (75.8%) achieved apCR. The tpCR rates were 73.1%, 52.1%, and 65.1% in the TcbHP, THP, and AC-THP groups, respectively. The bpCR and apCR rates were 79.3% and 80.7%, 60.6% and 71.3%, and 79.4% and 71.4% in the TcbHP, THP, and AC-THP groups, respectively. According to the MP grading system, 85.1% (n = 257) of the patients achieved G4 to G5. Three treatment regimens were significantly different with respect to tpCR (*P* = .004) and bpCR (*P* = .003), but not in apCR (*P* = .166) (Table 2).

**Table 2:** Evaluation of the curative effect of neoadjuvant therapy schemes (N [%]).

	Total	TcbHP	THP	AC-THP	_χ_^2^	*P*
	N = 302	N = 145	N = 94	N = 63		
**MRI**					11.874	.018
** CR**	179 (59.3)	97 (66.9)	43 (45.7)	39 (61.9)		
** PR**	98(32.5)	37 (25.5)	40 (42.6)	21 (33.3)		
** SD**	25(8.3)	11 (7.6)	11 (11.7)	3 (4.8)		
**tpCR**					11.016	.004
** tpCR**	196 (64.9)	106 (73.1)	49 (52.1)	41 (65.1)		
** non-tpCR**	106 (35.1)	39 (26.9)	45 (47.9)	22 (34.9)		
**bpCR**					11.612	.003
** bpCR**	222 (73.5)	115 (79.3)	57 (60.6)	50 (79.4)		
** non-bpCR**	80 (26.5)	30 (20.7)	37 (39.4)	13 (20.6)		
**apCR**					3.597	.166
** apCR**	229 (75.8)	117 (80.7)	67 71.3)	45 (71.4)		
** non-apCR**	73 (24.2)	28 (19.3)	27 (28.7)	18 (28.6)		
**MP**					15.718	.015
** 2**	5 (1.7)	3 (2.1)	2 (2.1)	0 (0.0)		
** 3**	40 (13.2)	16 (11.0)	20(21.3)	4 (6.3)		
** 4**	35 (11.6)	11 (7.6)	15 (16.0)	9 (14.3)		
** 5**	222 (73.5)	115 (79.3)	57 (60.6)	50 (79.4)		
**RCB**					12.779	.047
** 0**	196 (64.9)	106 (73.1)	49 (52.1)	41 (65.1)		
** 1**	47 (15.6)	18 (12.4)	18 (19.1)	11 (17.5)		
** 2**	47 (15.6)	16 (11.0)	21 (22.3)	10 (15.9)		
** 3**	12 (4.0)	5 (3.4)	6 (6.4)	1 (1.6)		

apCR = axillary pathological complete response, bpCR = breast pathological complete response, MP = Miller-Payne, MRI = magnetic resonance imaging, PD = progressive disease, PR = partial response, RCB = residual breast cancer burden, SD = stable disease, tpCR = total pathological complete response.

### 3.3. Related factors influencing the tpCR

**Figure 2.**
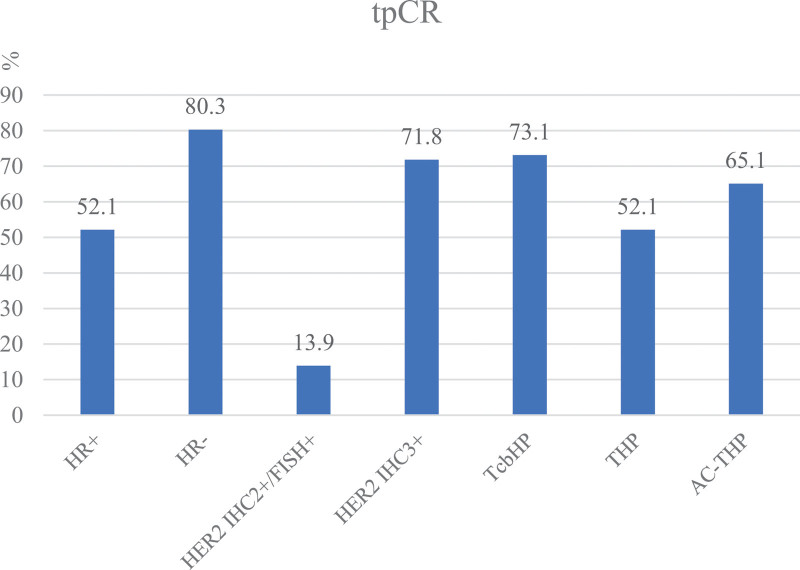
HR, HER2 status, treatment regimens and the tpCR rate of patients with HER2-positive breast cancer treated with neoadjuvant therapy .HER2 = human epidermal growth factor receptor 2, HR = hormone receptor, pCR = pathological complete response.

**Figure 3.**
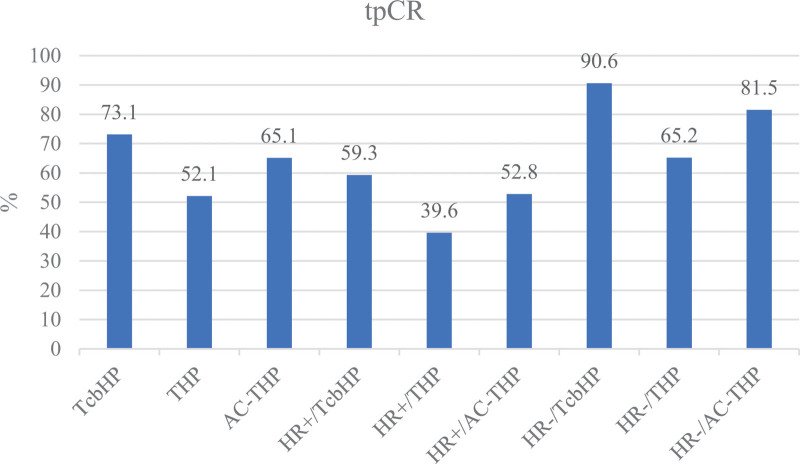
HR status and different regimens on the tpCR rate of patients with HER2-positive breast cancer treated with neoadjuvant therapy .HER2 = human epidermal growth factor receptor 2, HR = hormone receptor, tpCR = total pathological complete response.

According to the univariate analysis, HR, Her-2 status, and treatment regimen were significant predictors of tpCR (Table 3 and Fig.2). According to the multivariate logistic regression analysis, HR, Her-2 status, and treatment regimen were also independent predictors of tpCR (Table 4). The tpCR rate of the HR-negative group (80.3%) was higher than that of the HR-positive group (52.1%) and the difference was significant (*P* < .001, OR:3.505 [95%CI:1.981–6.202] in the multivariate). Patients with HER-2 IHC 3 + tumors had better pCR than patients with HER2 IHC 2+/FISH + tumors (71.8% vs 13.9%, *P* < .001, OR:11.958 [95%CI:4.342–32.931] in the multivariate). The tpCR rates in TcbHP and AC-THP were superior to those in THP. The tpCR rates in the HR-negative tumors were 90.6% with the TcbHP regimen, 65.2% with THP and 81.5% with AC-THP (*P* = .007), respectively. The tpCR rates in the HR-positive groups were 59.3% with the TcbHPregimen, 39.6% with THP and 52.8% with AC-THP (*P* = .100), respectively (Fig. 3).

**Table 3:** Univariate analysis of influence factors for the tpCR rate of patients with HER2-positive breast cancer treated with neoadjuvant therapy (N [%]).

Characteristics	tpCR	non-tpCR	OR(95%CI)	*P*
**Age (years**)			1.005 (0.625–1.616)	.983
≤** 50**	89 (65.0)	48 (35.0)		
** >50**	107 (64.8)	58 (35.2)		
**Menopause**			0.737 (0.459–1.185)	.208
** No**	85 (61.2)	54 (38.8)		
** Yes**	111 (68.1)	52 (31.9)		
**cT**			1.410 (0.849–2.343)	.185
** cT1-2**	142 (67.3)	69 (32.7)		
** cT3-4**	54 (59.3)	37 (40.7)		
**cN**			2.422 (0.793-7.393)	.120
** cN0**	17 (81.0)	4 (19.0)		
** cN1-3**	179 (63.7)	102 (36.3)		
**Clinical stage**			1.076 (0.666–1.739)	.765
** II**	83 (65.9)	43 (34.1)		
** III**	113 (64.2)	63 (35.8)		
**Histological classification**			1.106 (0.533–2.295)	.788
** G2**	174 (65.2)	93 (34.8)		
** G3**	22 (62.9)	13 (37.1)		
**HR**			0.267 (0.159–0.449)	<.001
**+**	86 (52.1)	79 (47.9)		
–	110 (80.3)	27 (19.7)		
**HER2**			0.063 (0.024–0.169)	<.001
** IHC 2+/FISH +**	5 (13.9)	31 (86.1)		
** IHC 3+**	19 1(71.8)	75 (28.2)		
**Ki-67**			0.638 (0.190–2.142)	.467
** ≤14%**	6 (54.5)	5 (45.5)		
** >14%**	190 (65.3)	101 (34.7)		
**Scheme**				.005
** TcbHP**	106 (73.1)	39 (26.9)	2.496 (1.445–4.311)	.001
** THP**	49 (52.1)	45 (47.9)	1.712 (0.887–3.303)	.109
** AC-THP**	41 (65.1)	22 (34.9)		
**HR+**				.100
** TcbHP**	48 (59.3)	33 (40.7)	2.220 (1.071–4.601)	.032
** THP**	19 (39.6)	29 (60.4)	1.706 (0.712–4.086)	.231
** AC-THP**	19 (52.8)	17 (47.2)		
**HR–**				.007
** TcbHP**	58 (90.6)	6 (9.4)	5.156 (1.828–14.537)	.002
** THP**	30 (65.2)	16 (34.8)	2.347 (0.747–7.374)	.144
** AC-THP**	22 (81.5)	5 (18.5)		

AC-THP = anthracycline + cyclophosphamide sequentially taxane, or docetaxel + H + P, FISH = fluorescence in situ hybridization, HER2 = human epidermal growth factor receptor 2, HR = hormone receptor, IHC = immunohistochemistry, TcbHP = taxane or docetaxel + carboplatin + trastuzumab(H) + pertuzumab (P), THP = taxane or docetaxel + H + P, tpCR = total pathological complete response.

**Table 4:** Multivariate analysis of influence factors for the rate of tpCR of patients with HER2-positive breast cancer treated with neoadjuvant therapy.

Characteristics	OR	95%CI	*P*
HR (– vs +)	3.505	1.981–6.202	<.001
HER2 (IHC 3 + vs IHC 2+/FISH+)	11.958	4.342–32.931	<.001
Scheme			.009
(TcbHP vs THP)	2.608	1.405–4.844	.002
(AC-THP vs THP)	2.023	0.950–4.307	.068

AC-THP = anthracycline + cyclophosphamide sequentially taxane, or docetaxel + H + P, FISH = fluorescence in situ hybridization, HER2 = human epidermal growth factor receptor 2, HR = hormone receptor, IHC = immunohistochemistry, TcbHP = taxane or docetaxel + carboplatin + trastuzumab(H) + pertuzumab (P), THP = taxane or docetaxel + H + P, tpCR = total pathological complete response

### 3.4. AEs

CTCAE5.0 was used for evaluating AEs. In the three regimens, grade 3 and 4 toxicities occurred with TcbHP more than those with THP and AC-THP. The most frequent serious AEs were leukopenia, neutropenia, and thrombocytopenia. Other common adverse effects were nausea, vomiting, asthenia, and diarrhea. The dose of neoadjuvant treatment was adjusted following the occurrence of AE in eleven patients treated with TcbHP. Most of the AEs in AC-THP and THP were of grades 1 to 2. Cardiac toxicity (grade 1) was reported in six patients with AC-THP, five with TcbHP, and three with THP regimens. No toxicity leading to death was reported (Table 5).

**Table 5:** Adverse effects related to neoadjuvant therapy in patients with HER2-positive breast cancer (N [%]).

Adverse effects	TcbHP (%) N = 145		THP (%) N = 94		AC-THP (%) (N = 63)	
	1–2	3–4	1–2	3–4	1–2	3–4
**Nausea**	62 (42.8)	7 (4.8)	29 (30.9)	3 (3.2)	23 (36.5)	3 (4.8)
**Vomiting**	62 (42.8)	7 (4.8)	29 (30.9)	3 (3.2)	22 (35.0)	3 (4.8)
**Anorexia**	57 (39.3)	5 (3.4)	35 (37.2)	0 (0.0)	20 (31.7)	0 (0.0)
**Insomnia**	45 (31.0)	4 (2.8)	23 (24.5)	0 (0.0)	17 (27.0)	0 (0.0)
**Fatigue**	60 (41.4)	4 (2.8)	31 (33.0)	0 (0.0)	23 (36.5)	0 (0.0)
**Muscle pain**	52 (35.8)	4 (2.8)	23 (24.5)	0 (0.0)	14 (22.2)	0 (0.0)
**Bone pain,**	45 (31.0)	2 (1.4)	20 (21.3)	0 (0.0)	15 (23.8)	0 (0.0)
**Diarrhea**	31 (21.4)	2 (1.4)	11 (11.7)	0 (0.0)	14 (22.2)	0 (0.0)
**Constipation**	24 (16.6)	2 (1.4)	11 (11.7)	0 (0.0)	15 (23.8)	0 (0.0)
**Oral ulcer**	53 (36.6)	0 0.0)	22 (23.4)	0 (0.0)	18 (28.6)	0 (0.0)
**Limbs numb**	27 (18.6)	0 (0.0)	17 (18.1)	0 (0.0)	11 (17.5)	0 (0.0)
**Cardiac function decline**	5 (3.4)	0(0.0)	3 (3.2)	0 (0.0)	6 (9.5)	0 (0.0)
**Leukopenia**	50 (34.5)	14 (9.7)	21 (22.3)	3 (3.2)	17 (27.0)	3 (4.8)
**Neutropenia**	50 (34.5)	14 (9.7)	21 (22.3)	4 (4.3)	17 (27.0)	3 (4.8)
**Thrombocytopenia**	38(26.2)	16(11.0)	5(5.3)	0(0.0)	4 (6.3)	1 (1.6)

HER2 = human epidermal growth factor receptor 2.

## 4. Discussion

HER2-positive breast cancer, a type of breast cancer with a high risk of recurrence and metastasis, has attracted increasing attention. The pCR rates in clinical neoadjuvant trials ranged from 39% to 66% with pertuzumab and trastuzumab in combination with different chemotherapy drugs.^[6,11,12,15,16]^ The 5-year follow-up data in the NeoSphere trial published in 2016^[17]^ showed that the dual-target neoadjuvant therapy was superior to a single-target regimen and confirmed that the tpCR was related to long-term survival. The Germany WSG ADAPT study^[18]^ showed that the pCR rate was 90% in HER2-positive/HR-negative tumors receiving neoadjuvant trastuzumab, pertuzumab, and 12 weeks of paclitaxel chemotherapy. The KRISTINE trial^[7]^ confirmed that the 3-year follow-up data showed that pCR was related to the decline of invasive DFS.^[19]^

A real-world study from Spain (NEOPETRA)^[20]^ included a total of 243 patients with HER2-positive breast cancer treated with HP plus chemotherapy as neoadjuvant therapy. In that study, the tpCR rate was 66.4%, 71% with anthracyclines and taxanes, 59.3% with single agent taxane, and 48.6% with platinum-based combinations. In the present study, the pCR rates in different treatment regimen were different from those in the NEOPETRA trial. In our study, the tpCR rate in the TcbHP group was the highest (73.1%). The tpCR rates were 52.1% and 65.1% in the THP group and in the AC-THP group, respectively. Differences in the tpCR rate in three different treatment regimens were more significant in HR negative tumors (*P* = .007) but were not significant in HR positive tumors (*P* = .100). Joo Young Ha et al^[21]^ analyzed 172 patients with HER2-positive breast cancer receiving TcbHP as the neoadjuvant treatment regimens, and the pCR rate was 60.8%. Our study had much higher pCR rates in TcbHP regimens, and the tpCR rate in HR-negative tumors reached 90.6%.

No clear conclusion can be drawn about which kind of chemotherapy regimen combined with a dual-target regimen was the best. In our study, we found that treatment regimens influenced tpCR and bpCR but had smaller effects on apCR. Furthermore, dual target plus combined chemotherapy regimens (TcbHP and AC-THP) were better than single agent chemotherapy regimens (THP) in the rate of tpCR. A study from the United States^[22]^ revealed that the pertuzumab-based regimen (including AC-THP densely dosed, 60%; TCHP, 63%; and THP, 55%) had a higher pCR rate. THP resulted in significantly fewer cycle delays because of toxicity compared with the other regimens. THP also resulted in the least dose reductions, the lowest rate of hospitalization, and the lowest rate of treatment discontinuation. Our study showed that the tpCR rate was 52.1% in the THP regimen, lower than those in other combined chemotherapy regimens, but had fewer side effects, especially for older patients or patients with complications.

Our study found that HR was the independent predictor of tpCR. Patients with HR negative tumors achieved a higher tpCR rate (80.3%) than those with HR positive tumors (52.1%) and the difference was significant (*P* < .001, OR:3.505 [95%CI:1.981-6.202] in the multivariate). The low pCR rate in patients with HR positive tumors may be associated with mutual interference between the HER2 receptor and the HR receptor, causing resistance to anti-HER2 treatments.

We found that HER2 status was the key factor that influenced the pCR, and the pCR rate in HER2 IHC 3 + tumor was significantly better than that in HER2 IHC 2+/ FISH + tumor. Chen et al^[23]^ analyzed 62 received dual HER2 blockade and the pCR rate was higher in tumors with HER2 IHC 3 + than in tumors with HER2 IHC 2 +/ FISH + (71.4% vs 47.4%, *P* = .009). In the present study, we included more patients treated with dual HER2 blockade, and patients with HER-2 IHC 3 + tumors had better pCR than patients with HER2 IHC 2+/FISH + tumors (71.8% vs 13.9%, *P* < .001, OR:11.958 [95%CI:4.342-32.931] in the multivariate), and patients with HER2 IHC 2+/FISH + tumors had much lower pCR than that study.

This study found that the apCR rate can be as high as 75.8%. For this group of patients, the sentinel lymph node biopsy had a higher reliability regarding avoiding axillary lymph node dissection. This study also provides data supporting the use of sentinel lymph node biopsy after neoadjuvant therapy in clinical practice.

We included a small number of patients and followed a retrospective design. Therefore, the findings of this study must be validated in a larger sample for more reliable clinical data, and long-term follow-up is still needed to obtain survival data.

## 5. Conclusions

In conclusion, HP combined with chemotherapy as neoadjuvant therapy has achieved a higher pCR rate in Chinese patients with HER2-positive breast cancer in the real world. Among the three combination regimens, TcbHP had the highest pCR. HR, HER2 status and treatment regimen were independent predictors of tpCR. HR-negative tumors demonstrated a higher pCR than HR-positive tumors. Patients with HER-2 IHC 3 + tumors had better pCR than patients with HER2 IHC 2+/FISH + tumors. The adverse reactions of the regimens are controllable.

## Acknowledgments

The authors thank Chunxiao Li for the help in data collection.
